# Reconciling irrigated food production with environmental flows for Sustainable Development Goals implementation

**DOI:** 10.1038/ncomms15900

**Published:** 2017-07-19

**Authors:** Jonas Jägermeyr, Amandine Pastor, Hester Biemans, Dieter Gerten

**Affiliations:** 1 Research Domain Earth System Analysis, Potsdam Institute for Climate Impact Research (PIK), Telegraphenberg A62, 14473 Potsdam, Germany; 2 Geography Department, Humboldt-Universitä¤t zu Berlin, Unter den Linden 6, 10099 Berlin, Germany; 3 International Institute for Applied Systems Analysis, Schlossplatz 1, 2361 Laxenburg, Austria; 4 Water Systems and Global Change Group, Wageningen University & Research, PO Box 47, 6700 AA Wageningen, The Netherlands; 5 Laboratoire d'étude des Interactions Sol-Agrosystème-Hydrosystème, INRA-IRD-SupAgro, 2 place Viala, 34060 Montpellier, France; 6 Water and Food Group, Wageningen University & Research, PO Box 47, 6700 AA Wageningen, The Netherlands

## Abstract

Safeguarding river ecosystems is a precondition for attaining the UN Sustainable Development Goals (SDGs) related to water and the environment, while rigid implementation of such policies may hamper achievement of food security. River ecosystems provide life-supporting functions that depend on maintaining environmental flow requirements (EFRs). Here we establish gridded process-based estimates of EFRs and their violation through human water withdrawals. Results indicate that 41% of current global irrigation water use (997 km^3^ per year) occurs at the expense of EFRs. If these volumes were to be reallocated to the ecosystems, half of globally irrigated cropland would face production losses of ≥10%, with losses of ∼20–30% of total country production especially in Central and South Asia. However, we explicitly show that improvement of irrigation practices can widely compensate for such losses on a sustainable basis. Integration with rainwater management can even achieve a 10% global net gain. Such management interventions are highlighted to act as a pivotal target in supporting the implementation of the ambitious and seemingly conflicting SDG agenda.

## Abstract (web summary)

Sustainable development goals for water use and food production are in conflict, but this could be reduced by proper water management. Here, violations of global environmental flow requirements for rivers are quantified and related to reconciliation potentials in irrigated and rainfed agriculture.

Global agricultural intensification through ever-increasing resource use is a main driver of current transgressions of ‘planetary boundaries', that is, critical global and regional levels of anthropogenically influenced Earth system processes such as land use change, biodiversity loss, freshwater use and nitrogen and phosphorus loads^1^. Transgressing these planetary boundaries increases the risk that the Earth system is transformed into a post-Holocene state with characteristics that potentially undermine system resilience and human welfare^1^. Because agriculture is central to attaining the renewed sustainable development goals (SDGs), they acknowledge this risk by committing all countries to a bold and transformative agenda in support of the twin challenge: protection of Earth's life-support system while reducing hunger and poverty^2^. With the human population set to rise to at least nine billion by 2050, the implementation of this vision aligned with environmental guardrails requires precautionary policies based on solid quantitative grounds such as formulated in the planetary boundaries framework^3^. For progress monitoring, a global SDG indicator framework has been developed^4^, but proposed actionable specifications for environment-related indicators remain insufficiently advocated^4^,^5^.

Freshwater resources, as a core example, are over-exploited and aquatic ecosystems are thereby rapidly degrading in many regions^6^,^7^. Restoration of currently compromised river ecosystems through securing environmental flow requirements (EFRs)—that is, the daily river flow needed to maintain aquatic ecosystem services and, thus, the human livelihoods that rely on them^8^—would entail a substantial reduction in water availability for irrigated food production. Accounting for >70% of human water withdrawals, irrigation is globally the largest user of freshwater^9^. To quantitatively underpin water targets in the SDG framework (6.4 and 6.6 as specified below) that bridge ecosystem maintenance, sustainable water use and food production, this study demonstrates how heavily current irrigation practices rely on EFRs. We show how much of global food production would be affected if policies to secure EFRs were implemented worldwide in the vein of propositions in the Brisbane Declaration^8^ and other aquatic ecosystem policy recommendations^10^,^11^,^12^. Correspondingly, we quantify—towards the food production target 2.3—the degree to which effective farm water management can outweigh associated constraints on irrigated food production. Using EFRs as an indicator is compatible with the regional planetary boundary for human freshwater use that accounts for the spatial and temporal pattern of local tolerance levels of water use and their transgression^1^,^13^.

To approach such analyses at global scale we employ an advanced dynamic biosphere model that represents natural and agricultural vegetation with associated ecological, hydrological and biogeochemical processes—including river flows, here newly implemented EFR regulations, irrigation and crop production—in a single internally consistent framework at high spatio-temporal resolution^14^. Reflecting methodological uncertainty and varied policies concerning the fraction of river flow which should remain untouched, we apply three differing hydrological methods to allocate daily flow volumes to EFRs (VMF method as in Pastor *et al*.^15^ and adapted versions of Tessmann^16^ and Smakhtin *et al*.^17^). Simulations are performed for the time period 1980–2009, with and without consideration of EFRs. In the former case, water withdrawal for irrigation and other purposes (household, industry and livestock, HIL) is disallowed as long as it would tap EFRs. To put irrigation into perspective of total food production, we also illustrate a scenario with an absence of irrigation and highlight exemplary scenarios of moderate irrigation system upgrades and more integrated farm water management (overview of simulations in Table 1).

## Results

**Figure 1:**
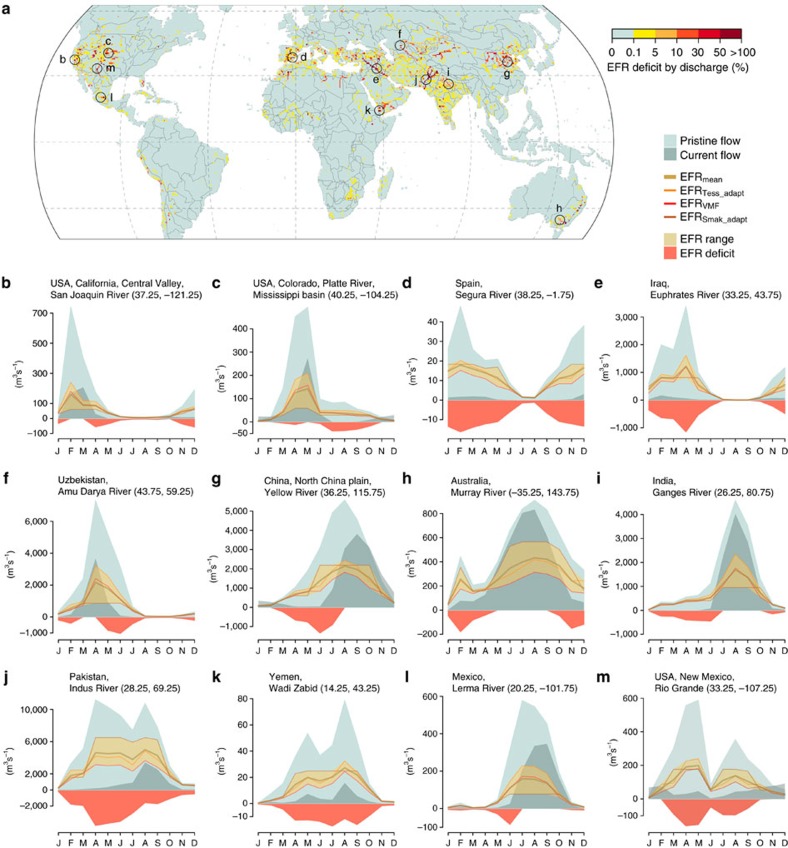
Discharge and environmental flows of selected river stretches. The map (**a**) illustrates the mean annual EFR deficit (EFR minus current discharge, if >0) relative to mean annual discharge. Hydrographs (**b**–**m**, locations indicated on the map) highlight seasonal flow alterations (pristine versus current discharge) together with EFR estimates (mean, Tessmann_adapted_, VMF, Smakhtin_adapted_, Methods section) and, if unmet, the mean EFR deficit. All estimates are in m^3^ s^−1^ and for the time period 1980–2009.

Our results show that today’s human water withdrawals, 2,409 km^3^ for irrigation and 1,071 km^3^ for HIL (1980–2009 average), harm many river stretches around the world. Figure 1 lays out regions and the degree to which EFRs are currently undermined to sustain the human water demand, which is the case especially in Central and South Asia, the North China plain, the Middle East, the Mediterranean region and North America (Fig. 1a). EFR transgressions reach a level beyond the uncertainty range in these regions and thus indicate severe degradation, given by the three estimation methods applied (Supplementary Fig. 1). Figure 1a illustrates the mean annual EFR deficit (EFR minus discharge, if >0) relative to current mean annual discharge. The Indus river in Pakistan represents a dramatic case, where this ratio exceeds 100% at annual level—that is, less than half of the needed environmental flows are currently available—while EFRs remain unmet throughout 11 months per year (Fig. 1j). Yet we also find alarming EFR violations along many other rivers such as the Amu Darya, Euphrates, Yellow River, Ganges, Murray and Rio Grande (Fig. 1b–m). Supplementary Fig. 2 details EFR transgressions in terms of the total annual deficit and the number of months with transgressions. 31% of global EFR deficits occur in Pakistan alone, reaching 58.4% together with India (17.7%) and China (9.7%). Global EFR deficits involve a water overuse of 997 km^3^ per year for irrigation (equalling 41% of total current irrigation water use) and a further 236 km^3^ per year (22%) for HIL (Table 2 and Supplementary Table 1). If not indicated otherwise, results refer to the mean of three EFR methods.

**Figure 2:**
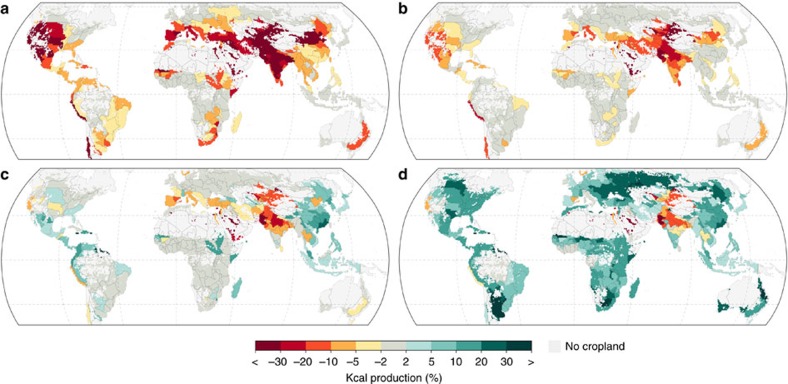
Governing environmental flows constrains food production. The maps illustrate the change in total (that is, rainfed and irrigated) kcal production in the absence of irrigation (**a**), with irrigation constrained by EFRs (mean of three EFR methods) (**b**), with upgraded irrigation constrained by EFRs (**c**) and with integrated water management constrained by EFRs (**d**), with respect to the current situation and aggregated to Food Production Units (1980–2009). Cells without significant cropland fraction (<0.1%) are masked.

**Figure 3:**
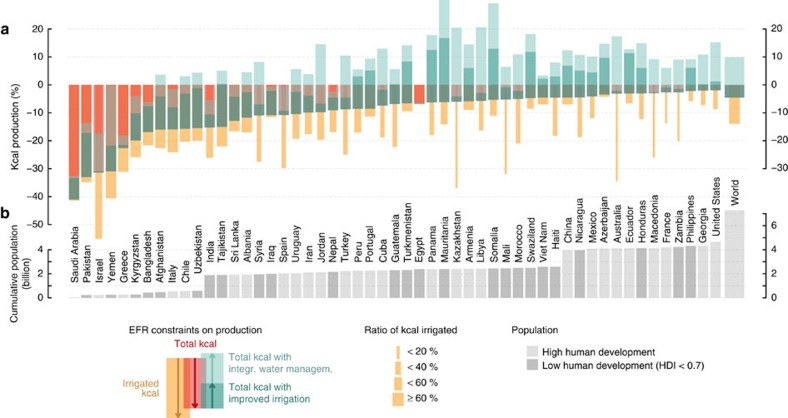
Reconciling EFRs and food production across countries. Countries are ordered by the dependence of kcal production on EFRs (red) (**a**). Beige bars highlight production declines on irrigated cropland only. Compensating effects of two different water management scenarios ('Scenario 3 irrigation upgrade' and 'scenario 4 integrated water management', Table 1) on total production are indicated in mint green. Cumulative country population is shown in the bottom **b**, distinguishing countries with a high or low Human Development Index (HDI)^18^ (details in Supplementary Table 2).

Current food production thus heavily relies on water that would actually be needed to sustain riverine ecosystems (Fig. 2). If EFRs were to be preserved—also in regions where irrigated food production currently depends on them, and without compensating water saving efforts—52% of global irrigated cropland would face kcal production losses ≥10% (Table 2). Among intensely irrigated regions, like many Mediterranean countries, the Middle East, North America and particularly parts of Central and South Asia, as much as >20% of total (that is, rainfed and irrigated) kcal production relies on EFRs—at the aggregated level of food production units (Fig. 2b). Figure 3 highlights the interaction of maintaining EFRs and agricultural production at country level—Saudi Arabia, Pakistan and Israel appear in first place (>30% total loss). But also India, Bangladesh, Uzbekistan, Afghanistan, Italy and Greece, among others, would face a production decline of ∼15–23% (Fig. 3, Supplementary Table 2). Global irrigated kcal production would be subjected to a 13.9% loss, corresponding to a 4.6% loss of total production (Table 2). This number is significant, given that irrigation water sustains only 15% of total global kcal production (while another 18% are sustained by precipitation on irrigated land, confirming earlier estimates^9^). It illustrates that maintaining EFRs would impinge on about a third of the current overall contribution to agricultural production made by irrigation—in the absence of water management improvements.

Countries where ≥10% of production relies on the withdrawal of EFRs are inhabited by 2.03 billion people; 90% of them live in countries with a Human Development Index^18^ <0.7 (Supplementary Table 2). Since agriculture is at the center of human development and poverty reduction, serious societal impacts are to be expected in default of other adaptation or compensation measures. Case study observations confirm complex difficulties in water re-allocation and infrastructure re-organization for ecosystem conservancy if environmental flows are tapped already^12^,^19^,^20^. Yet this is still a prerequisite if additional and sometimes irreversible degradation of aquatic ecosystems is to be avoided. EFR implementation is indivisibly linked to the achievement of stable and resilient food production systems, needed to ground nested environmental, social and economic sustainability (Supplementary Fig. 3)^3^,^21^,^22^.

Field-based and modelling studies indicate that management improvements can advance crop water productivity on a considerable scale^7^,^23^,^24^,^25^,^26^. To compensate for EFR constraints, we here develop an irrigation upgrade scenario as one example of a spectrum of effective farm water management options^14^. Our simulations suggest that a transition from surface to sprinkler irrigation systems (using half of thus saved consumptive losses for expansion, Table 1) would suffice—at global level—to outweigh kcal constraints associated with implementation of EFR policies (Table 2, Fig. 3). Irrigation requirements could thereby decrease to about half the current amount, mainly through reductions in conveyance losses and return flows. While meeting EFRs, irrigation water consumption (withdrawals minus return flows and drainage losses) remains at the same level (global average ∼35% below current value, Table 2), but with higher shares of productive crop transpiration, which reflects the increase in water productivity associated with the improved management^27^. Gains are naturally marginal in countries operating highly efficient systems already (for example, Israel), but major gains are possible in countries with presently predominately large-scale surface irrigation systems and unlined conveyance canals such as Pakistan, Afghanistan, Uzbekistan and Bangladesh (Fig. 3). In Tajikistan, for instance, EFR constraints on food production imply a 15% loss, but allied with improved irrigation systems a net gain of 0.4% could be reached, and in Turkmenistan a loss by 6.6% could be turned into an 8.4% gain (Supplementary Table 2).

Yet even under such improved irrigation water use, on 34% of irrigated cropland ≥10% of kcal production would still have to rely on EFRs, mostly in Central and South Asia, which is compensated at global level by production gains in other regions, notably in East Asia (Fig. 2c). More ambitious interventions are needed to reconcile irrigation requirements and EFRs in hot-spot regions such as the Aral Sea basin^28^. For example, the integration of rainwater management (water harvesting, mulching, conservation tillage) and irrigation upgrades are associated with sizeable potentials in many of these regions^14^. To explore this further, we also analyse an exemplary scenario of integrated farm water management, that is, combining above defined irrigation upgrades with rainwater management (25% of surface runoff collected for supplemental irrigation and 25% of soil evaporation alleviated, Table 1). Results indicate that, while overcompensating EFR-induced constraints across many countries (Figs 2d and 3), such comprehensive interventions could increase global total kcal production by 9.9% compared to the current situation (Table 2).

## Discussion

Overall, the water management strategies quantified here illustrate that opportunities to thrive do exist within planetary environmental guardrails^29^. While not exhaustive—only even more ambitious interventions presented in Jägermeyr *et al*.^14^ prove sufficient to halve the global food gap by 2050—they highlight that farm water management across scales, linked to preservation of environmental flows, would greatly assist the intricate task of such implementations paired with the goals of poverty reduction and agricultural productivity increase as outlined by the SDGs. Nevertheless, our spatially explicit simulations demonstrate that farm water management alone might not suffice to simultaneously achieve possibly conflicting SDG targets 6.4 (‘sustainable withdrawals') and 2.3 (‘double agricultural productivity') by 2030. Additional important merits, such as incorporating ecological landscape approaches including soil fertility optimization and advanced crop varieties will be needed to further maximize synergies and thus crop water productivity—promising examples have been demonstrated^30^,^31^,^32^.

**Figure 4:**
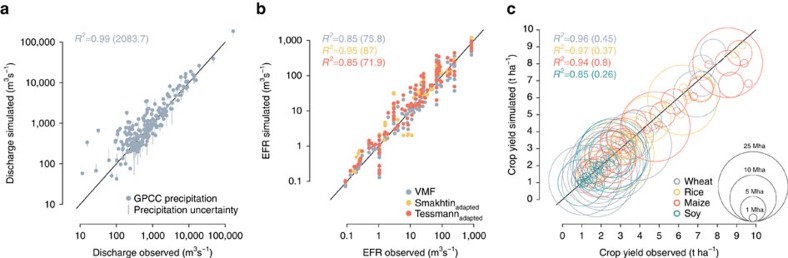
Model evaluation for the reference period 1980–2009. Validation against observational data is shown for mean annual discharge (uncertainty range from use of four precipitation data sets) (**a**), environmental flow requirements for three differing calculation methods (**b**) and country-level crop yields (calibrated for management intensity) for main staple crops and the respective top 30 producer countries, with chart symbols scaled by the country's cropland area (**c**). The coefficient of determination (*R*^2^) is shown along with the root mean square error in parenthesis (further details in Methods section 'Model validation').

Assessment of water saving and crop yield potentials in specific locations requires more in-depth studies of what methods are best suited in view of local biophysical conditions and social appropriateness. For many regions the scenarios imply major technological and also institutional transformations away from current less efficient and ecologically detrimental practices, and here suggested potentials may not be reached where such transformations will not, or only partially, be realised. Conversely, higher potentials appear feasible for regions where even more radical transformations may occur^14^,^27^. Water availability is estimated under the assumption that industrial and domestic water demand is prioritized over agricultural water demand, whereas constraints on irrigated production could further increase in regions where, for example, bioenergy production will impose new pressure on freshwater resources^33^, or where water quality or thermal pollution limits water use^34^. The hydrological EFR calculation approach builds on the best currently available methods and appears adequate as we incorporate three different, referenced EFR methods. Nevertheless, such first-order estimates need refinement to achieve more holistic quantifications of ecosystem water needs. The validated dynamic modelling capacity (Fig. 4 and Methods section ‘Model validation') well represents observed water stress signals in crop growth, while accounting for water trade-offs along the river network.

Despite substantial untapped farm-level management opportunities, institutional changes and revisions of current water concessions appear necessary in view of broader EFR implementations^19^,^28^. A number of local initiatives prove successful already^12^, for example, Uzbekistan has set clear policy targets for water use and savings and was able to reduce its water footprint^35^,^36^. The ‘redline’ water policies in China illustrate the integration of national legislation with local institutional frameworks^26^. With Australia, the United States and South Africa in the forefront, the validity of setting EFRs has become internationally accepted and in many countries provisions are being developed^8^,^10^,^11^,^12^,^37^. But the systematic and comprehensive quantification of EFRs poses methodological, institutional and financial challenges and is thus still insufficient. Together with often ineffective governance, this explains why existing licenses and policies are not yet being implemented^12^,^38^, although it is clear that EFR assessment and regulation should be a basic requirement of Integrated Water Resource Management, as, for example, outlined in the EU Water Framework Directive^39^ and now the SDG 6.5. That said, recognising the environment as a legitimate user of water still has not led to the institutional reforms needed to ensure environmentally sustainable basin management in competition with other water users like agriculture and industry^40^.

As yet, there is no evidence base to evaluate quantitatively SDG target interactions^22^. The current lack of established tools and thresholds to quantify the SDG water agenda^41^ forms a barrier to translate the agreed principles into concrete action. For example, the indicator for sustainable freshwater withdrawals (6.4.2) was proposed to be directly linked to the EFR concept, but ultimately not stipulated^4^. Through a transparent and consistent approach that is suited for global applications and dynamically couples processes relating EFRs to crop production, this study adds quantitative evidence that the ambitious targets of SDG 2 (food security) and 6 (environmental sustainability) could be met to a large extent through improved water management alone. We point out that the critical reinforcing interaction of water productivity increases (6.4) with both sustainable withdrawals (also part of target 6.4) and agricultural productivity (2.3) benefit from simultaneous implementation pathways (also closely related to targets 2.4 and 6.6, detailed in Supplementary Fig. 3)^21^. In view of the multifaceted SDG target interactions^42^, integrated strategies of improved farm water management appear central. However, associated opportunities, for instance rainwater harvesting, that are coupled to vital socio-economic and environmental co-benefits especially for smallholders^32^,^43^, have not gained required international attention among high-level development policies^44^.

While the here adopted first-order quantifications of EFRs require local refinements^12^,^40^,^45^, they are key to transboundary trade-off analyses beyond the conceptual stage and highlight current pressure on freshwater resources that is to be overcome by more sustainable farming systems. In conclusion, our study suggests that achieving the ambitious commitments of the SDG agenda—while staying within the safe operating space of the Earth system as delineated by the planetary boundaries—appears feasible, but remains a grand implementation challenge.

## Methods

### Environmental flow requirement objectives

We estimate EFRs, irrigation demand and withdrawals and crop calorie production with a biosphere model that simulates these processes daily, as an intrinsic part of natural and managed ecosystem dynamics. We use the concept of EFRs to allocate maximum allowed monthly water withdrawals, expressed as a percentage of ‘pristine' undisturbed mean monthly river flow (determined globally for each 0.5° grid cell from a simulation without considering human land use, water infrastructure and water withdrawals; forced with climate data of the simulation period 1980–2009; see below). We include three hydrological EFR estimation methods to depict an uncertainty range, which reflects methodological differences and which can be interpreted as the outcome of different environmental policies. Based on a simulation considering current agricultural patterns, reservoir management and multi-sectoral human water withdrawals (see ‘Model and simulation protocol' below), this uncertainty range is also used to classify river segments according to the current status of transgression of EFRs, that is, the sub-global freshwater use boundary^1^ (Supplementary Fig. 1).

The EFR calculation methods aim at reaching a ‘fair' ecological status, which is a conservative assumption as this status can still be characterized by disturbed biota, loss or reduction in spatial distribution of sensitive species and occurrence of alien species^17^. The Tessmann method^16^ and the Variable Monthly Flow (VMF) method^15^ account for seasonal EFR variation by distinguishing high-, intermediate- and low-flow regimes based on different proportions of mean monthly river flow (MF) and mean annual flow (AF) of long-term average ‘pristine' conditions (Supplementary Table 3). To protect habitat maintenance and essential flow variability, supporting the ‘natural flow paradigm'^46^, for each month different flow volumes are allocated to EFRs based on the flow regime (Supplementary Table 3). In this study we use an adapted version of Tessmann's method (hereinafter labelled Tessmann_adapted_). We replace the most restrictive parameter that allocates 100% of river flow during low flow periods by more approved 80%, a value proposed by Richter *et al*.^47^, which has been employed in other studies^48^.

The Smakhtin *et al*.^17^ method comprises two components, a minimum baseflow (exceeded 90% of the time, Q_90_) and a percentage of AF depending on mean seasonal river flow variability. For rivers with stable seasonal flow and thus high Q_90_ values relative to AF (Q_90_>30% AF), only the baseflow is allocated. In cases of higher flow variability (Q_90_ can go down to zero for intermittent rivers), fractions of AF are allocated additionally (*h* in Supplementary Table 3). The Smakhtin *et al*. method provides by definition static EFR targets throughout the year, which contradicts the ‘natural flow paradigm' and has thus been criticized^47^,^49^. To beneficially employ a quantile method, we use an adapted version of the Smakhtin *et al*. approach (hereinafter labelled Smakhtin_adapted_) and allow for seasonal variation. As proposed and tested in Pastor *et al*.^15^, we combine it with the Q_90_/Q_50_ method and thereby replace the Q_90_ baseflow by Q_50_ during high flow periods (Supplementary Table 3). In addition, we restrict EFR allocations to not exceed 80% of monthly pristine river flow.

Due to the characteristics of a quantile method, the Smakhtin_adapted_ method is the least strict method applied in this study at river stretches with highly variable flow regimes, and compares as the strictest method at stable flow regimes. The Tessmann_adapted_ method is generally stricter than the VMF method—hydrographs in Fig. 1 compare all three methods. Overall, such conceptually simple ‘per cent of flow' approaches can be seen as first-order proxies for EFR estimates, based on the hydrologic regime. While more comprehensive approaches including other ecological indicators are required for developing local river planning targets, such hydrologic methods are important for tradeoff analysis applicable at large scales and are shown to provide already a high degree of protection for natural flow variability when implemented^37^,^47^.

### Model and simulation protocol

The LPJmL model globally represents biogeochemical land surface processes, simulating daily water fluxes in direct coupling with the establishment, growth and productivity of major natural and agricultural plant types at 0.5° resolution^27^,^50^. Crop production is represented by 12 specified crop functional types, irrigated or rainfed. Spatially explicit data on cropland extent is obtained from the MIRCA2000 land use data set^51^ and the extent of areas equipped for irrigation from Siebert *et al*.^52^. A recently implemented mechanistic irrigation module provides the framework for irrigation transitions and the spatially explicit distribution of irrigation systems^27^. Land use patterns are held constant at year 2005.

Carbon assimilated through photosynthesis is allocated to harvestable storage organs (for example, cereal grain) and three other pools (roots, leafs and stems). Sowing dates are calculated based on climate and crop type, but fixed during the simulation period after 1980. In tropical regions that exhibit predominant precipitation seasonality, sowing dates on irrigated land are forced to occur in the dry season. All simulations run for the time period 1980–2009, and LPJmL is forced with the Climate Research Unit’s (CRU) TS 3.1 monthly climatology for temperature, cloudiness^53^ and with the Global Precipitation Climatology Centre’s (GPCC) precipitation data^54^. Model simulations follow a 900-year (no land use) and 120-year (land use) spin-up recycling the first 20 years of input climatology.

A simulation omitting human water use is performed based on the same land use patterns, but under rainfed conditions only (‘1. No irrigation', Table 1). Otherwise gross irrigation requirements are mechanistically calculated based on local crop-specific water demand and irrigation system application requirements^27^. Thermoelectric water demand related to cooling water requirements for industrial processes and electricity production, but also household and livestock production requirements (HIL), are implicitly considered from an external source^55^. Total withdrawal requirements (that is, irrigation and HIL) are constrained by local availability of renewable freshwater, including a representation of dams and reservoirs^56^ with daily EFR release regime (yet channel and habitat maintenance floods not considered). This setup is referred to as ‘current situation' (Table 1).

Precipitation and irrigation water is partitioned into plant transpiration, soil evaporation, interception loss, surface and subsurface runoff and deep percolation, in direct coupling to daily weather conditions and the surface, soil water and energy balance^27^. Surface and subsurface runoff are accumulated along the river network and subsequently available for downstream reuse. In this study there is no implicit assumption about fossil groundwater abstraction and water diversions, which are expected to amount to ∼20% of global irrigation water requirements^57^. While our EFR estimates thus do not account for fossil groundwater contributions, such flows are not directly relevant for the feedback of surface freshwater EFRs and food production.

On the basis of these validated streamflow estimates (see Fig. 4 and section ‘Model validation' below), EFRs are calculated as described above. In the ‘2. Respect EFR' simulation (Table 1), total water withdrawal is temporally restricted as long as it would tap EFRs, while industrial and domestic withdrawals are prioritized over irrigation withdrawals. For each above-defined EFR method we perform an individual model run, but results presented throughout the text refer to the mean of the three simulations and the s.d. is assigned in Table 2 (individual results are shown in Supplementary Table 1).

In view of supporting EFR implementations, we simulate additional scenarios of improved farm water management. We present a moderate scenario of irrigation system upgrades (‘3. Respect EFR paired with irrigation upgrade', Table 1), combined with EFR constraints from the 2. scenario. Surface irrigation systems are assumed to be replaced by sprinkler systems—except paddy rice, which remains with surface systems throughout all irrigation scenarios—and half of saved consumptive ‘losses' are assumed to be made available to expand irrigation into neighbouring rainfed cropland, while the total cropland area remains constant^14^. Since observed efficiency improvements do not necessarily result in lower water withdrawals as farmers often expand irrigation or use higher value crops, instead of losing water allocations^58^, we allocate half of saved consumptive water to irrigation expansion if rainfed cropland is available in the same grid cell. Note that return flows are not considered savable losses throughout this study as they might be accessible for downstream users.

Finally, as an outlook towards more comprehensive approaches, we present a scenario with a modest form of integrated farm water management, that is, irrigation upgrades from Scenario 3 combined with rainwater management, yet paired with EFR constraints (‘4. Respect EFR paired with integrated farm water management', Table 1). Rainwater management in this simulation comprises collecting 25% of surface runoff for supplemental irrigation during dry spells and alleviating 25% of soil evaporation (for example, mulching, conservation tillage). Jägermeyr *et al*.^14^ provides additional modelling details and also a wider spectrum of more ambitious farm water management interventions.

### Model validation

The LPJmL model has been validated extensively in terms of biogeochemical, ecological and hydrological processes for both natural and agricultural systems^59^,^60^,^61^,^62^,^63^. It has the unique feature to simulate vegetation dynamics and the carbon and water cycle in a single consistent framework and therefore bridges categories between a global gridded crop model and a global hydrological model^64^. LPJmL explicitly considers human interventions such as irrigation and reservoir operation, and it solves the irrigation water balance in a mechanistic way, for all major crop types and with considerable spatial and temporal detail—the cornerstone for reasonable simulations of irrigation system transitions, water savings potentials and interaction of irrigation water constraints with food production^27^.

A validation of LPJmL-simulated key variables for the time period 1980–2009 is highlighted in Fig. 4. Uncalibrated LPJmL mean annual discharge simulations are compared with the latest observations from GRDC (Global Runoff Data Centre) stations^65^ (Fig. 4a). We select discharge stations for which more than 100 months of observations are available during the simulation period, and where the mismatch between the reported and simulated contributing area is ≤15% (*n*=203). In general, observed discharge is well simulated across most stations (*R*^2^=0.99), yet at some stations we find non-marginal biases. Recent global hydrological model intercomparison projects confirm that LPJmL is among the state-of-the-art models and LPJmL's predictive uncertainty is in line with competing models^64^,^66^,^67^. While model comparisons reveal significant uncertainties associated with individual parameterizations, large parts of predictive uncertainty in discharge simulation can be attributed to the differences in precipitation data^61^,^68^. To address this issue here, we ground the validation of discharge simulations in four different precipitation data sets (GSWP3 (ref. 69), PGFv2.1 (ref. 70), WATCH+WDFEI^71^ and GPCC^54^, the latter employed throughout this study). Figure 4a shows that gauge observations mostly fall into the uncertainty range stemming from different inputs.

Hydrological EFR estimations applied globally in this study are validated against 11 local case study estimates situated across 5 Major Habitat Types (a classification of freshwater ecoregions based on Abell *et al*.^72^) and a wide range of river flow and climate regimes across North America, South America, Europe, Africa and Asia. Incorporated case studies provide 10–50 years of daily flow observations and present a spectrum of different EFR estimation methods. These include hydrological methods (similar to those employed in this study), hydraulic methods (defining flow velocity and river cross-section for fish habitat maintenance), habitat-simulation methods (based on the relationship between salmon survival monitoring and flow quantity), and comprehensive holistic methods (based on ecological monitoring and expert judgement)^15^. The comparison of LPJmL-simulated EFR estimates with estimates based on local observations reveals good agreements (Fig. 4b), the coefficient of determination (*R*^2^) ranges between 0.85 and 0.95 for all three methods (VMF, Tessmann_adapted_, Smakhtin_adapted_). Pastor *et al*.^15^ acknowledge that the VMF method generally performs best for large-scale modelling applications. This validation captures a sufficiently broad range of environmental settings to support the application of all three methods at the global scale.

Average country-level crop yield simulations, calibrated to emulate the effect of current management practices^50^, exhibit high agreement with observations obtained from FAOSTAT^73^ (Fig. 4c). The *R*^2^ across top 30 producer countries varies between 0.85 and 0.97 for the four major staple crops (wheat, rice, maize and soy). In absolute terms, simulated global kcal production of 7.8 × 10^15^ kcal in year 2006 is ∼18% short of reported values (9.5 × 10^15^ kcal)^74^, mostly because LPJmL currently does not account for multi-cropping systems. LPJmL simulations show the capacity to explain a high fraction of observed inter-annual yield variability among the important producer countries (Supplementary Fig. 4). LPJmL's sensitivity to crop water stress, fundamental for reproducing yield variability, is verified in the following sensitivity analysis. In a simulation assuming that water demand of irrigated and rainfed crops is to be always met throughout the growing season—that is, water stress effects on crop growth are circumvented — explained yield variability decreases strongly for the range of investigated sites compared to the standard LPJmL simulation and turns statistically insignificant (*P*≥0.1). This effect appears especially pronounced for wheat (Supplementary Fig. 4a–c), somewhat less distinct for maize (Supplementary Fig. 4d–f) and disappears for rice (Supplementary Fig. 4g–i). These different levels of sensitivity can be explained by the fact that maize as a C4 crop is generally less sensitive to drought stress using a more efficient enzyme on the pathway of CO_2_ fixation^75^, while rice (mostly paddy rice) is assumed to be provided with sufficient soil moisture among the countries presented in Supplementary Fig. 4. Overall, Supplementary Fig. 4, but also the broader benchmarking evaluation in the context of Global Gridded Crop Model Intercomparison (GGCMI)^63^,^76^ provide evidence for LPJmL's capability of representing most relevant mechanisms of climate-induced signals in observed yields, in particular those linked to water stress. This is key to explaining inter-annual yield variability and eventually feedbacks from water management on food production levels.

A number of case studies are in line with our findings. For instance, a recent study^19^ focussed on the Guadiana basin in Spain quantifies implications of maintaining environmental flow requirements to meet ‘good ecological status' of rivers as required by the European Water Framework Directive (WFD)^39^. Results indicate that irrigation water supplies could be affected by up to 40% (we simulate 41% at global level, Table 2), translating into 20% reduction in farmers' income. In the Murray-darling basin, maintaining EFRs might lead to a net irrigation revenue decrease by 2.4% per year, if no compensating measures were implemented^77^. On the basis of the (modified) VMF method, another recent study^78^ supports our results in that efficiency improvements in farm water management might not suffice to meet EFRs in hot spot regions (study is focussed on Lake Urmia, Iran), and more drastic measures, such as revisions of water concessions, might be needed in the future. Safeguarding EFRs can, however, also increase downstream water availability and thus farmers' income^79^. At global scale, yet with an overly simple approach, it has been shown that maintaining EFRs might pose a greater challenge for global irrigation water availability than climate change-related alterations of the discharge regime^80^.

### Data availability

The LPJmL code and data supporting the findings of this study are available from the corresponding author on request.

## Additional information

**How to cite this article:** Jägermeyr, J. *et al*. Reconciling irrigated food production with environmental flows for Sustainable Development Goals implementation. *Nat. Commun.* **8,** 15900 doi: 10.1038/ncomms15900 (2017).

**Publisher’s note:** Springer Nature remains neutral with regard to jurisdictional claims in published maps and institutional affiliations.

## Supplementary Material

Supplementary Information

Peer Review File

## Figures and Tables

**Table 1:** Simulation protocol.

**Scenario**	**Description**
0. Current situation	Water withdrawals constrained by surface water availability only, not by EFRs.
	
1. No irrigation	No human water withdrawal (irrigation and other sectors), but same land use patterns as in 'current situation', that is, rainfed conditions on currently irrigated cropland.
	
2. Respect EFR	Total water withdrawal constrained by EFRs (industrial and domestic prioritized over irrigation). Individual model runs for each EFR method. (Tessmann_adapted_, VMF, Smakhtin_adapted_).
	
3. Respect EFR paired with irrigation upgrade	Same as scenario 2, yet combined with an irrigation upgrade scenario: surface irrigation replaced by sprinkler systems (except paddy rice), half of saved consumptive losses used to expand irrigation into neighbouring rainfed cropland. Individual model runs for each EFR method.
	
4. Respect EFR paired with integrated water management	Same as scenario 3, yet combined with a modest form of rainwater management: 25% of surface runoff collected for supplemental irrigation (rainwater harvesting) and 25% of soil evaporation alleviated. Individual model runs for each EFR method.

The table highlights scenarios investigated in this study to quantify environmental flow requirements (EFRs) and potentials in farm water management. The simulation setup is detailed in the Methods section 'Model and simulation protocol'.

**Table 2:** Agricultural impacts through water conservation and management.

**Scenario**	**Total kcal production**	**Irrigated kcal production**	**Total area affected (kcal loss≥10%)**	**Irrigated area affected (kcal loss≥10%)**	**Irrigation water withdrawal**	**Irrigation water consumption**
	**(% change)**	**(% change)**	**(%)**	**(%)**	**(% change)**	**(% change)**
1. No irrigation	−14.7	−44.4	32.5	81.3	−100.0	−100.0
2. Respect EFR	−4.6 (±0.8)	−13.9 (±2.5)	16.1 (±1.8)	52.2 (±3.9)	−41.4 (±5.8)	−35.1 (±5.6)
3. Respect EFR with irrigation upgrade	−0.1 (±1.0)	5.6 (±2.9)	12.0 (±2.4)	33.6 (±7.4)	−54.4 (±4.3)	−34.8 (±5.2)
4. Respect EFR with integrated water management	9.9 (±1.0)	6.8 (±2.9)	8.2 (±2.0)	30.5 (±7.5)	−55.7 (±4.3)	−36.8 (±5.2)

Change in global kcal production and the proportion of affected area (kcal loss ≥ 10%) is shown for the total absence of irrigation (1.), irrigation constrained by environmental flow requirements (EFRs) (2.), upgraded irrigation constrained by EFRs (3.) and integrated water management constrained by EFRs (4.)—all compared to the current situation (1980–2009). Scenario setups are detailed in Table 1. Also listed are associated changes in irrigation water withdrawal (IWD) and consumption (IWC). Note that kcal production and area affected refer to cropland area, while IWD and IWC refer to the total irrigated area (incl. cash crops, cotton and so on). Precipitation still partly sustains production on irrigated land in 1. 2–4. refer to the mean of three EFR methods (with s.d. in parentheses), Supplementary Table 1 presents respective absolute values.
