# Supplementary material for: Reconciling irrigated food production with environmental flows for Sustainable Development Goals implementation
Source: Nat Commun. 2017 Jul 19;8:15900. doi: 10.1038/ncomms15900 (PMC5524928; doi:10.1038/ncomms15900)
Supplement: Supplementary Information [file ncomms15900-s1.pdf]

Type of file: PDF
Size of file: 0 KB
Title of file for HTML: Supplementary Information
Description: Supplementary Figures, Supplementary Tables and Supplementary References

Type of file: PDF
Size of file: 0 KB
Title of file for HTML: Peer Review File
Description:

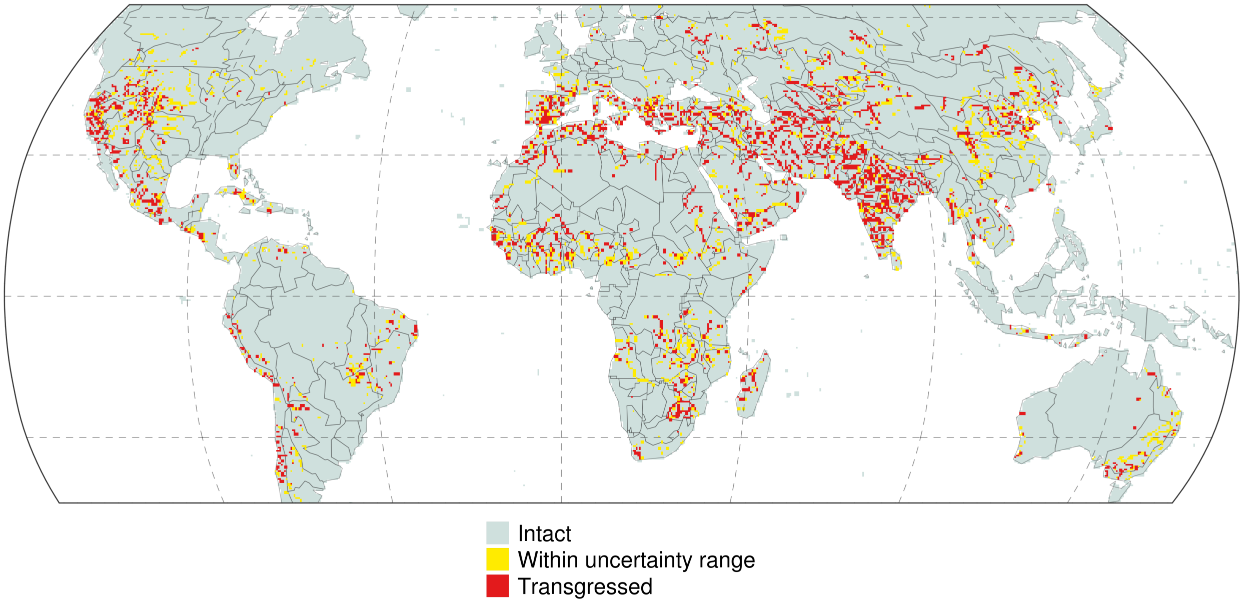

Supplementary Figure 1: Uncertainty range of the current status of environmental flow transgressions. The degree to which EFRs are undermined is expressed as the transgression-to-uncertainty ratio (>5% 'Within uncertainty range', >100% 'Transgressed'), averaged over months with EFR transgressions (1980–2009, 0.5° resolution). Borders delineate Food Production Units. The proportion of EFR deficit (EFR_{def}) and the EFR uncertainty (range of EFR estimates from three methods as defined in the Methods section), calculated for each month and grid cell (EFR_{status}). EFR_{status} is shown as the average over months in which both pristine river discharge and current EFR_{def} are ≥ 0.1 m^3s^{-1}, throughout the simulation period 1980–2009. $EFR_{def} = max(\text{EFR} - \text{current discharge}, 0)$ is calculated as the mean of the three EFR methods. The map in Figure 1a illustrates the proportion of mean annual EFR_{def} and current mean annual discharge. See Supplementary Figure 2a for the sum of annual EFR_{def} in million m^3 and Supplementary Figure 2b for the average number of months in which at least one of three methods indicates $EFR_{def} \geq 0.1$ m^3s^{-1}.

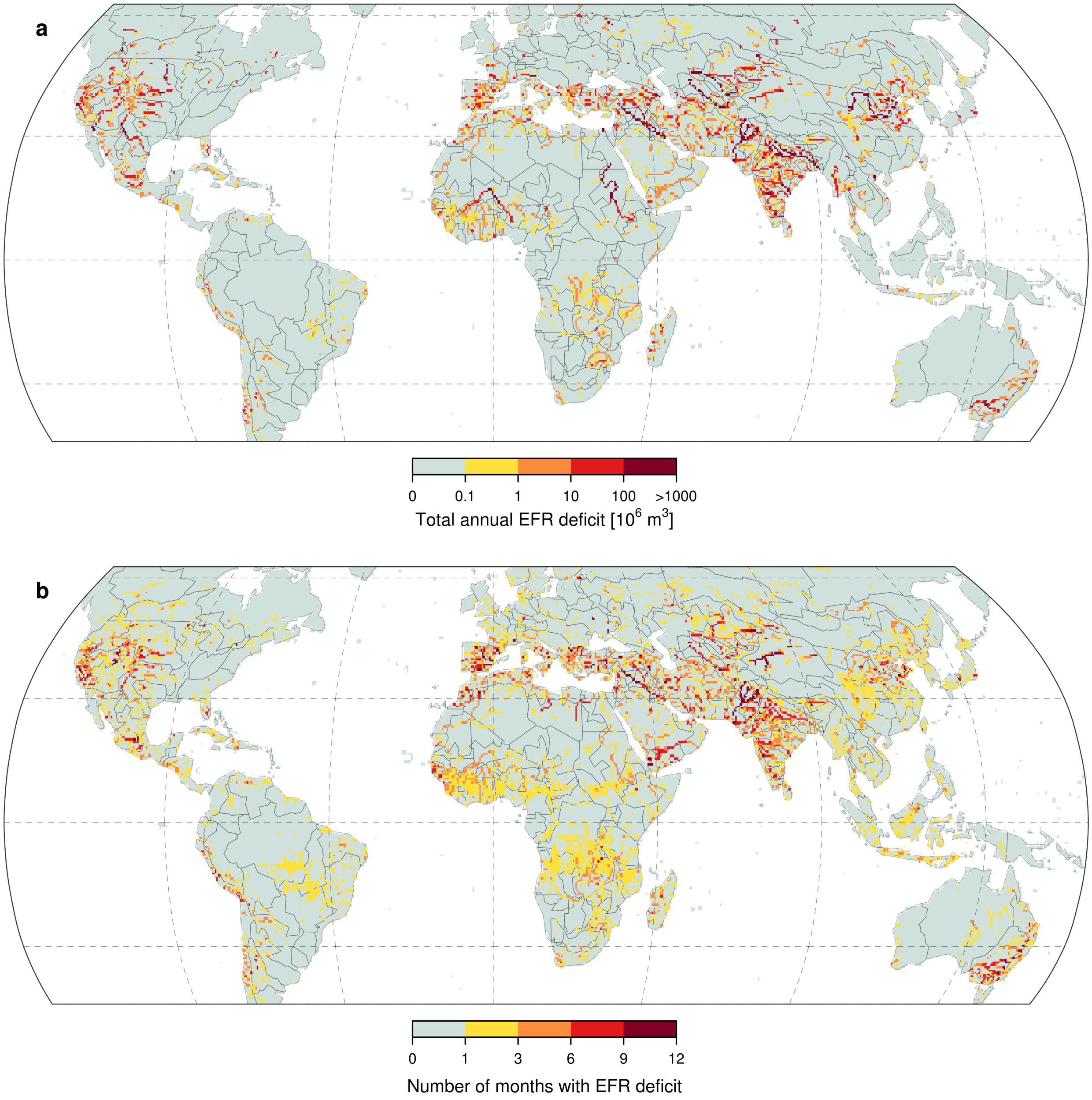

Supplementary Figure 2: Current status of environmental flow transgressions in absolute terms. Map (**a**) illustrates the total annual EFR transgressions in million m^3 (mean of three EFR methods), map (**b**) shows the number of months per year in which at least one of the three EFR methods indicates an EFR transgression > 0.1 m^3s^{-1} (1980–2009, 0.5° resolution).

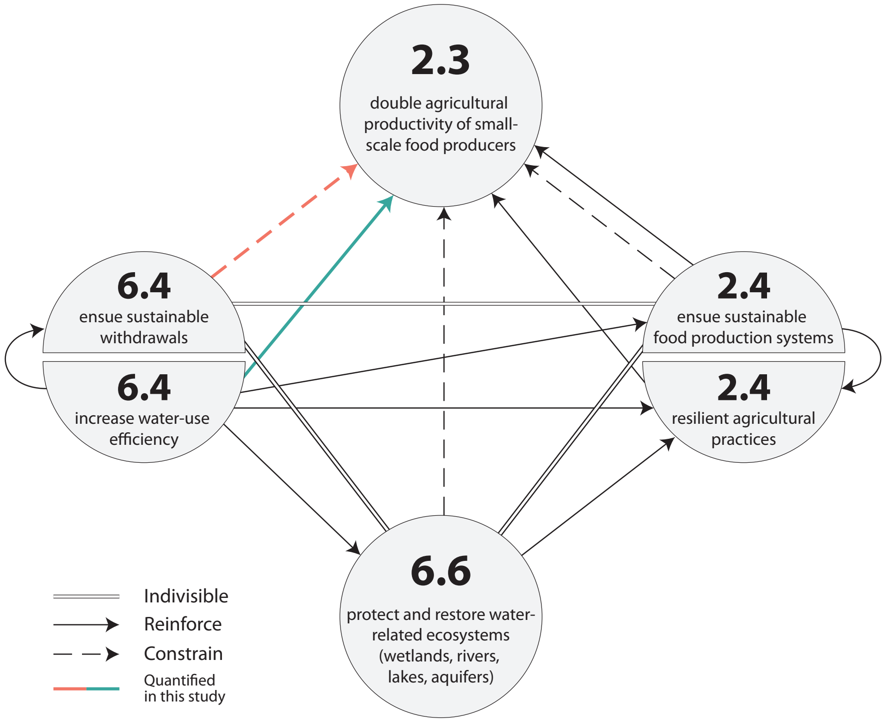

Supplementary Figure 3: Interaction of SDG targets subjected in this study. Constraints of maintaining sustainable withdrawals (6.4) on agricultural productivity (2.3) (red) and reinforcements of increased water-use efficiency (6.4) for 2.3 (mint) are explicitly quantified in this study. While in this context additional inter-linkages exist[1] (especially with SDG 15), the figure illustrates the most important interactions between SDG 2 and 6.

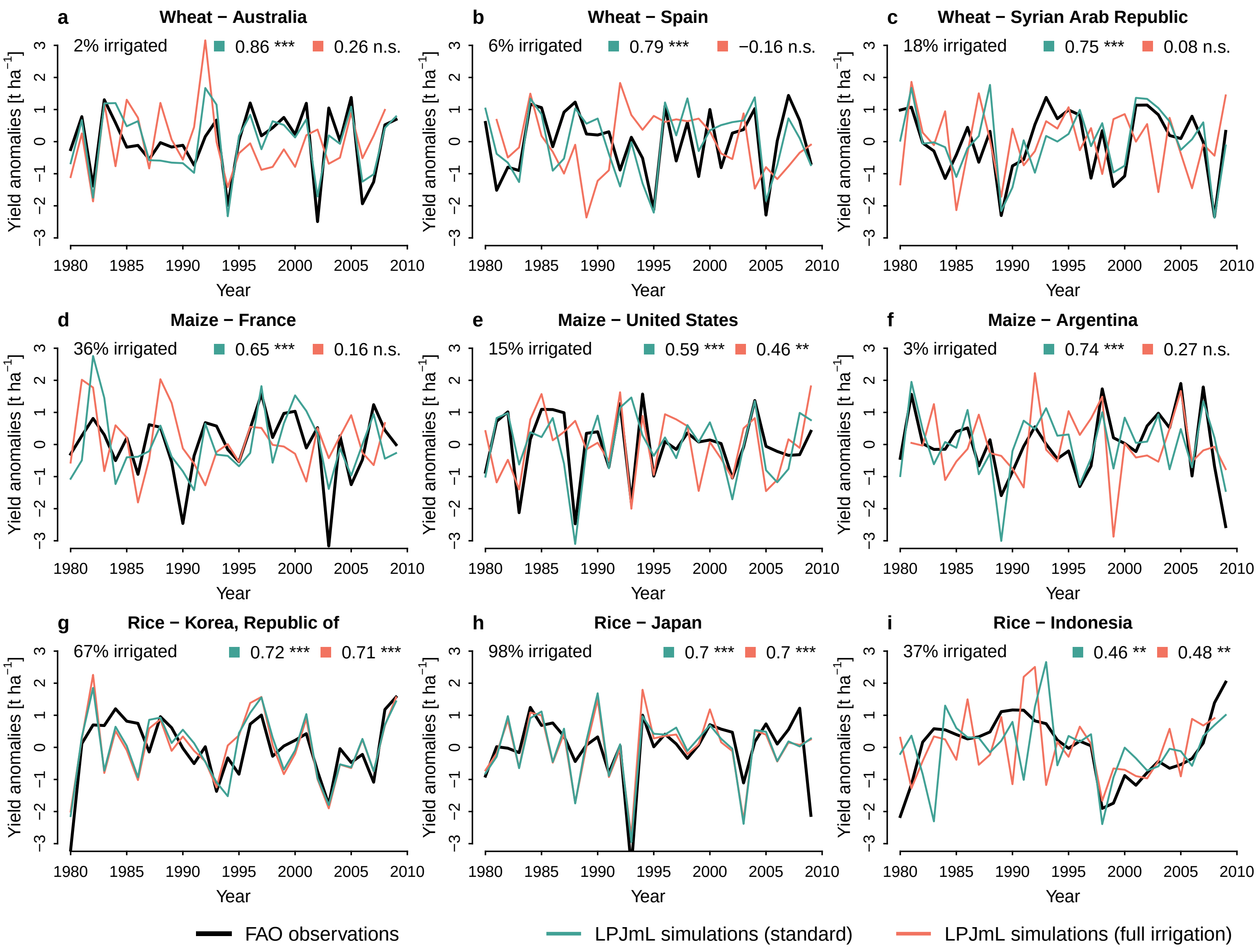

Supplementary Figure 4: Evaluation of observed and LPJmL-simulated yield variability. Country-level time series of detrended yield anomalies from FAOSTAT reference data[2] opposed to LPJmL simulations for wheat (**a–c**), maize (**d–f**) and rice (**g–i**). LPJmL simulations are shown for the standard simulation (irrigation constrained by surface water availability, mint) and a simulation which omits water stress for irrigated and rainfed crops (salmon). The Pearson's correlation coefficient is indicated respectively in the top right corner of each plot (significance: ***, p <0.001; **, p <0.05; *, p <0.1; n.s., not significant) and the fraction of cropland under irrigation in the top left corner.

Supplementary Table 1: Global sums of food production and water abstractions for different management scenarios. Global sums of kcal production, area affected (kcal loss > 10%), irrigation water withdrawal (IWD) and consumption (IWC) and withdrawal and consumption for household, industry and livestock (HIL_{WD} and HIL_{WC}) are shown for the following scenarios: current situation, i.e. irrigation without EFR constrains (**0.**), the absence of irrigation (**1.**), irrigation constrained by EFRs (**2.**), upgraded irrigation constrained by EFRs (**3.**), integrated water management constrained by EFRs (**4.**). 2.–4. are shown for three EFR methods, respectively. The simulation period is 1980–2009, see Table 1 for an overview of the simulation protocol.

	Total production [10^{13} kcal]	Irrigated production [10^{13} kcal]	Total area affected [Mha]	Irrig. area affected [Mha]	IWD [km^3]	IWC [km^3]	HIL_{WD} [km^3]	HIL_{WC} [km^3]
**0. Current situation**	740.0	244.2	0.0	0.0	2409.3	1254.7	1070.5	192.8
**1. No irrigation**	631.6	135.8	262.1	157.2	0.0	0.0	1,090.8	196.5
**2. Respect EFR**								
$Tessmann_{adapted}$	700.8	205.0	145.7	108.7	1305.5	756.8	802.5	143.6
VMF	712.6	216.7	116.2	93.3	1570.1	891.9	909.4	163.3
$Smakhtin_{adapted}$	704.7	208.8	128.8	102.2	1361.3	794.1	790.2	141.6
**3. Respect EFR with irrigation upgrade**								
$Tessmann_{adapted}$	732.9	251.7	117.5	81.2	1017.0	761.3	797.7	142.4
VMF	746.9	265.7	79.8	53.2	1214.7	889.7	904.2	162.0
$Smakhtin_{adapted}$	737.7	256.4	92.2	62.0	1065.0	801.6	784.8	140.1
**4. Respect EFR with integrated water management**								
$Tessmann_{adapted}$	807.3	254.5	84.4	74.9	986.7	737.5	792.5	141.8
VMF	821.2	268.4	53.7	46.9	1183.5	864.8	902.3	161.9
$Smakhtin_{adapted}$	812.1	259.3	60.8	55.0	1034.6	777.4	777.6	139.4

Supplementary Table 2: Country-level aggregation of changes in *kcal* production under EFR constraints and management improvements. Numbers in this table are illustrated in Figure 3. Columns indicated with '% change' refer to the change from the current situation. 'Ratio of kcal irrigated' is the ratio of irrigated production to total production. Population data are derived from UN Population Division[3] and Human Development Index (HDI) data from UNDP[4], who define values <0.7 as medium to low human development. Countries with <0.01 Mha irrigated cropland or <1 million population are omitted.

	**Scenario 2** **Respect EFR**			**Scenario 3** **Respect EFR + irrigation upgrade**		**Scenario 4** **Respect EFR + integr. water management**	**Scenario 0** **Current situation**		
	Total kcal	Irrigated kcal	Irrigated cropland	Total kcal	Irrigated cropland	Total kcal	Ratio of kcal irrigated	Population 2015	HDI
	[% change]	[% change]	[Mha]	[% change]	[Mha]	[% change]	[%]	[million]	[0,1]
Saudi Arabia	-41.03	-41.43	0.33	-33.45	0.37	-32.51	99	32	0.84
Pakistan	-33.01	-34.80	10.37	-17.16	11.41	-13.60	95	194	0.54
Israel	-31.40	-55.10	0.02	-30.84	0.02	-17.44	57	8	0.89
Yemen	-30.94	-40.59	0.32	-21.68	0.39	-0.33	76	28	0.50
Greece	-22.60	-31.10	0.38	-21.50	0.40	-18.09	73	11	0.86
Kyrgyzstan	-19.90	-25.88	0.31	-10.21	0.34	-4.10	77	6	0.66
Bangladesh	-16.91	-21.61	4.46	-7.60	5.31	-6.21	78	159	0.57
Afghanistan	-16.03	-22.60	1.38	-4.13	1.66	3.62	71	36	0.46
Italy	-15.94	-24.09	1.62	-8.05	1.96	-1.59	66	61	0.87
Chile	-15.71	-20.30	0.39	-3.13	0.47	3.14	77	18	0.83
Uzbekistan	-15.48	-19.98	1.00	-1.13	1.20	4.35	77	30	0.68
India	-15.29	-26.10	52.59	-10.57	62.32	-5.48	59	1307	0.61
Tajikistan	-15.02	-22.01	0.16	0.36	0.19	5.05	68	7	0.62
Sri Lanka	-12.89	-16.58	0.43	-4.22	0.50	-0.19	78	22	0.76
Albania	-11.73	-17.01	0.14	-2.65	0.16	4.54	69	3	0.73
Syria	-10.95	-27.56	0.77	-6.96	0.82	8.18	40	23	0.59
Iraq	-10.85	-11.55	1.75	-2.12	1.88	-0.67	94	36	0.65
Spain	-10.83	-29.82	1.21	-9.23	1.35	-0.36	36	47	0.88
Uruguay	-10.45	-19.29	0.18	-2.50	0.21	5.66	54	3	0.79
Iran	-9.88	-17.66	3.93	-3.85	4.42	3.88	56	79	0.77
Jordan	-9.75	-19.57	0.01	-6.66	0.01	14.58	50	7	0.75
Nepal	-9.05	-16.92	1.07	-4.57	1.31	-2.40	53	33	0.55
Turkey	-8.75	-24.88	2.52	-4.52	2.96	10.46	35	77	0.76

Peru	-8.61	-17.05	0.60	3.02	0.80	5.55	50	31	0.73
Portugal	-8.56	-11.31	0.26	5.14	0.32	9.36	76	11	0.83
Cuba	-7.41	-18.79	0.56	-1.71	0.70	12.98	39	11	0.77
Guatemala	-6.90	-22.24	0.11	0.26	0.14	5.63	31	17	0.63
Turkmenistan	-6.59	-9.44	0.48	8.37	0.58	14.13	70	5	0.69
Egypt	-6.50	-6.50	1.52	-6.91	1.55	-6.80	100	88	0.69
Panama	-6.31	-17.94	0.03	12.51	0.05	17.82	35	4	0.78
Mauritania	-6.20	-14.10	0.01	16.76	0.02	30.33	44	4	0.51
Kazakhstan	-6.00	-36.91	0.65	-4.12	0.74	20.40	16	17	0.79
Armenia	-5.86	-8.91	0.11	6.03	0.13	14.49	66	3	0.73
Libya	-5.74	-16.30	0.08	-2.87	0.09	20.65	35	7	0.72
Somalia	-5.35	-11.06	0.10	12.93	0.15	29.18	48	11	0.35
Mali	-5.23	-31.94	0.18	-0.86	0.25	6.48	16	18	0.42
Morocco	-5.05	-20.92	0.73	-2.11	0.84	10.93	24	34	0.63
Swaziland	-4.76	-8.54	0.03	11.82	0.04	18.24	56	1	0.53
Viet Nam	-4.58	-7.04	3.18	2.23	3.80	3.35	65	94	0.67
Haiti	-4.58	-18.16	0.10	3.00	0.15	8.00	25	11	0.48
China	-4.58	-7.01	50.67	6.88	63.19	12.40	65	1367	0.73
Nicaragua	-4.50	-18.69	0.07	5.10	0.10	10.78	24	6	0.63
Mexico	-4.15	-11.92	3.82	4.45	5.06	10.12	35	120	0.76
Azerbaijan	-3.45	-4.25	0.63	9.67	0.73	14.75	81	10	0.75
Australia	-3.17	-34.38	0.56	-2.14	0.68	17.36	9	24	0.94
Ecuador	-3.13	-6.62	0.42	11.32	0.57	12.74	47	16	0.73
Honduras	-3.11	-12.27	0.05	6.10	0.07	14.94	25	8	0.61
Macedonia	-2.99	-26.07	0.02	-2.68	0.02	9.24	11	2	0.75
France	-2.68	-13.77	1.90	-0.91	2.37	6.11	19	64	0.89
Zambia	-2.67	-20.09	0.04	-1.41	0.04	9.04	13	15	0.59
Philippines	-2.18	-5.79	1.37	6.14	1.81	9.22	38	101	0.67
Georgia	-2.04	-7.22	0.09	0.26	0.10	10.86	28	4	0.75
United States	-1.94	-8.69	13.84	1.27	16.69	15.26	22	322	0.92
World	-4.59	-13.93	193.40	-0.11	234.35	9.93	33	7269	0.71

Supplementary Table 3: Definition of hydrological seasons and respective EFR allocations. Mean monthly flow (MF), mean annual flow (AF) refer to pristine river flows, Q_{90} (Q_{50}) defines the base flow that is on average exceeded 90% (50%) of the time (simulated under 1980–2009 climate but in the absence of human water flow and land-use alterations). The Variable Monthly Flow method (VMF) is used following its original formulation[5] and modified versions of the Tessmann and Smakhtin et al. methods are used as described in the Methods section 'Environmental flow requirement objectives'.

**EFR method**	**Flow regime classification**		**Environmental flow requirements**		
	low-flow	high-flow	low-flow	intermediate-flow	high-flow
$\text{Tessmann}_{\text{adapted}}$	$\text{MF} \leq 40\%\ \text{AF}$	$\text{MF} > \text{AF}$	$80\%\ \text{MF}$	$40\%\ \text{AF}$	$40\%\ \text{MF}$
VMF	$\text{MF} \leq 40\%\ \text{AF}$	$\text{MF} > 80\%\ \text{AF}$	$60\%\ \text{MF}$	$45\%\ \text{MF}$	$30\%\ \text{MF}$
$\text{Smakhtin}_{\text{adapted}}$	$\text{MF} \leq 80\%\ \text{AF}$	$\text{MF} > 80\%\ \text{AF}$	$Q_{90} + h$	–	$Q_{50} + h$
				$h = \begin{cases} 0, & \text{if } Q_{90} > 30\%\ \text{AF} \\ 7\%\ \text{AF}, & \text{if } Q_{90} \leq 30\%\ \text{AF} \\ 15\%\ \text{AF}, & \text{if } Q_{90} \leq 20\%\ \text{AF} \\ 20\%\ \text{AF}, & \text{if } Q_{90} \leq 10\%\ \text{AF} \end{cases}$ $\text{EFR} = min(\text{EFR}, 80\%\ \text{MF})$	

Supplementary References

1. UN-Water. Water and Sanitation Interlinkages across the 2030 Agenda for Sustainable Development. Tech. Rep., UN Water, Geneva (2016).

2. FAO. FAOstat, Food and Agricultural Organization, Rome (2012). http://faostat3.fao.org/home/index.html (*accessed* 2016-06-18).

3. United Nations. World Population Prospects: The 2015 Revision. Tech. Rep., United Nations, Department of Economic and Social Affairs, Population Devision (2015).

4. UNDP. Human Development Report 2015. Tech. Rep., United Nations Development Programme (UNDP), New York, NY, USA (2015).

5. Pastor, a. V., Ludwig, F., Biemans, H., Hoff, H. & Kabat, P. Accounting for environmental flow requirements in global water assessments. *Hydrology and Earth System Sciences* **18**, 5041–5059 (2014).
