# Supplementary material for: Reconciling irrigated food production with environmental flows for Sustainable Development Goals implementation
Source: Nat Commun. 2017 Jul 19;8:15900. doi: 10.1038/ncomms15900 (PMC5524928; doi:10.1038/ncomms15900)
Supplement: Peer Review File [file ncomms15900-s2.pdf]

Editorial Note: this manuscript has been previously reviewed at another journal that is not operating a transparent peer review scheme. This document only contains reviewer comments and rebuttal letters for versions considered at Nature Communications.
